# Epigallocatechin-3-Gallate Reduces Neuronal Apoptosis in Rats after Middle Cerebral Artery Occlusion Injury via PI3K/AKT/eNOS Signaling Pathway

**DOI:** 10.1155/2018/6473580

**Published:** 2018-03-25

**Authors:** Wang Nan, Xu Zhonghang, Chen Keyan, Liu Tongtong, Guo Wanshu, Xu Zhongxin

**Affiliations:** ^1^Department of Neurology, China-Japan Union Hospital of Jilin University, Changchun 130033, China; ^2^Department of Neurology, The People's Hospital of Liaoning Province, No. 33 Wenyi Road, Shenyang, Liaoning 110016, China; ^3^Department of Laboratory Animal Science, China Medical University, No. 77 Puhe Road, Shenyang North New Area, Shenyang, Liaoning 110122, China

## Abstract

**Background/Aims:**

Epigallocatechin-3-gallate (EGCG) has neuroprotective effects and the ability to resist amyloidosis. This study observed the protective effect of EGCG against neuronal injury in rat models of middle cerebral artery occlusion (MCAO) and investigated the mechanism of action of PI3K/AKT/eNOS signaling pathway.

**Methods:**

Rat models of permanent MCAO were established using the suture method. Rat behavior was measured using neurological deficit score. Pathology and apoptosis were measured using HE staining and TUNEL. Oxidative stress and brain injury markers were examined using ELISA. Apoptosis-related proteins and PI3K/AKT/eNOS signaling pathway were determined using western blot assay and immunohistochemistry.

**Results:**

EGCG decreased neurological function score, protected nerve cells, inhibited neuronal apoptosis, and inhibited oxidative stress injury and brain injury markers level after MCAO. EGCG reduced the apoptotic rate of neurons, increased the expression of Bcl-2, and decreased the expression of Caspase-3 and Bax. After LY294002 suppressed the PI3K pathway, the protective effect of EGCG decreased after administration of PI3K inhibitors.

**Conclusion:**

EGCG has a protective effect on rat brain injury induced by MCAO, possibly by modulating the PI3K/AKT/eNOS signaling pathway.

## 1. Introduction

Ischemic stroke, the most common type of stroke, accounts for 60%–80% of all strokes [1]. Ischemic stroke has the characteristics of high incidence, high disability rate, high recurrence rate, and high mortality rate and has a trend of younger onset. In the early stage of ischemic stroke, the cerebral blood flow in the ischemic center drops sharply and neuronal cell death by necrosis occurs; however, the nerve cells around the ischemic center (ischemic penumbra) still have metabolic activity, The death was mainly apoptosis, and its morphological features were nuclear pyknosis, membrane foaming, and the formation of apoptotic bodies. Its morphological features are nuclear pyknosis, cellular membrane foaming, and the formation of apoptotic bodies [2, 3]. Therefore, apoptosis plays an important role in the pathogenesis of acute ischemic stroke.

After cerebral ischemia, excitatory amino acid toxicity, free radicals and NO damage, and immune inflammatory reaction are the main factors that mediate neuronal apoptosis [4]. Mitochondrial-mediated apoptosis pathway plays an important role in cerebral ischemia-induced neurocyte apoptosis. When cells were stimulated by ischemia and hypoxia, mitochondrial membrane is depolarized; then the mitochondrial membrane potential decreased, leading to opening the permeability transition pore and cytochrome C and Caspase/Smac/DIABLO are released from the mitochondria, and, in the presence of dATP/ATP, the apoptotic bodies are synthesized with apoptosis activating factor (Apaf-1), cause activation of Caspase-9, downregulate Caspase-3, and led to cell apoptosis. At present, the regulatory role of phosphoinositide 3-kinase (PI3K)/serine-threonine protein kinase (Akt) signaling pathway in neuronal apoptosis after cerebral ischemia has attracted much attention, and 3-inositol phosphate dependent protein kinase-1 (PDK1) enhances its activity by phosphorylating Akt [4]. Phosphorylation of Akt and phosphorylation of nitric oxide synthase (NOS) are important ways for endothelial cells to synthesize NO [5]. According to the regulation of the expression, cells or tissue origin of the prototype enzyme, and the order of cloning, NOS is divided into three subtypes: neuronal NOS (nNOS), endothelial NOS (eNOS), and inducible NOS (iNOS). By selectively regulating the NOS of different subtypes, we can protect or injure the damaged tissues after ischemia reperfusion.

It has been reported that the drug inhibition and gene interferon therapy can improve experimental cerebral ischemia injury. Sung et al. [6] have been shown that the p35 can significantly improve X-gal positive nerve cell survival in the ischemic penumbra and inhibit the expression level of Caspase-3 and Cyt C, and the CrmA group has increased neuronal survival, but no significant difference. Matsuura et al. [7] and other studies have found that the effective PARP-1 inhibitor MP-124 can reduce the area of cerebral infarction by 64% in the monkey MCAO model, which can significantly reduce the neurobehavioral score. Currently, more and more researches concern the potential therapeutic effects of tea polyphenols on senescence and brain aging. Tea polyphenols are rich in green tea, which accounts for 30%–40% of the dry weight of tea leaves. Tea polyphenols mainly contain four kinds of monomers: epigallocatechin-3-gallate (EGCG), epicatechin (EC), epigallocatechin (EGC), and (-)-epicatechin-gallate (ECG). EGCG content is the highest in tea, and its biological activity is the strongest [8]. Clinical and epidemiological studies have shown that green tea extract EGCG has neuroprotective effects on nerve repair, which has been confirmed from cell culture to animal models [9, 10]. Tea polyphenols are believed to have multifunctional neuroprotective effects, such as antioxidant activity [11], free radical scavenger [12], and iron chelating agent [13]. Haque et al. [14] and other studies suggested that the antioxidant activity of EGCG plays an important role in improving the cognitive ability of mice in space learning. This study established rat models of middle cerebral artery occlusion (MCAO) injecting with EGCG using arterial pump, observed neurological function and brain injury, established neuronal cell injury model of cerebral ischemia and hypoxia, and investigated the regulatory mechanisms of PI3K/AKT/eNOS signaling pathway* in vivo* and* in vitro*.

## 2. Materials and Methods

### 2.1. Experimental Animals and Group Assignment

Forty male Sprague-Dawley rats weighing 260–280 g were purchased from Laboratory Animal Department of China Medical University, China. This study has been approved by the Laboratory Animal Welfare and Ethics Committee of China Medical University (approval number: IACUC NO. 2015047). Rats were randomized into sham surgery group (sham group; *n* = 15), permanent MCAO group (MCAO + DMSO group; *n* = 15), MCAO + EGCG group (EGCG group; *n* = 15), and MCAO + EGCG + PI3K inhibitor LY294002 group (PI3K group; *n* = 15).

### 2.2. Establishment of Rat Models of MCAO

The rats were anesthetized with 2% sodium pentobarbital (6900083, Solarbio, Beijing, China), subjected to tracheal intubation, connected with ventilator and anesthesia machine, and anesthetized with Isoflurane (201705134, Shenzhen reward Life Technology Co. Ltd.). The rats lay in the supine position. The external carotid artery, its branches occipital artery, and superior thyroid artery were dissociated and the branches were ligated. Afterwards, external carotid artery was isolated towards the distal end. The common carotid artery and internal carotid artery were temporarily occluded with an artery clamp. The distal end of external carotid artery was occluded, and external carotid artery was cut off. The external carotid artery was folded back, and its stump was in a straight line with the internal carotid artery and inserted into the suture/microtube device (PE-0402 tube, Ningbo Anne Software Technology Co., Ltd.). Subsequently, the artery clamp was unclamped. The depth of suture insertion was 17–19 mm from the bifurcation of common carotid artery. The insertion was stopped when encountering resistance. Blood flow dropped to less than 30% of baseline revealed by transcranial laser Doppler flowmetry indicating successful establishment of MCAO models. At 2 hours after MCAO, the suture was pulled out. Blood flow restored to more than 80% of baseline revealed by transcranial laser Doppler flowmetry suggested successful reperfusion.

### 2.3. Dosage and Method of Administration

The rats in the EGCG group were intraperitoneally injected with EGCG (Sigma-Aldrich., 93894, USA) 20 mg/kg after MCAO. The sham groups were injected with an equal volume of saline after received the same surgical procedures except inserting a nylon filament. In the PI3K group, after anesthesia, the rat head was fixed in the stereotactic apparatus. Stereotaxic localization of lateral ventricles is as follows: 0.9 mm lateral to and 1.5 mm posterior to skull fontanel point (Bregma point). A hole was drilled with mini dental drill. 10 mmol/L LY294002 (Sigma-Aldrich, L9908, USA) solution 10 *μ*l was infused with a microsyringe at the depth of 3.8 mm. The fusion was finished within 5 minutes. The needle was maintained in place for 5 minutes. The spillage of blood, drugs, and cerebrospinal fluid was observed at the site of injection. The scalp was sutured. After injection in the lateral ventricle, MCAO was performed, and then EGCG 20 mg/kg was intraperitoneally injected.

### 2.4. Triphenyltetrazolium Chloride Solution (TTC) Staining

Five rats' heads were removed after phenobarbital sodium 40 mg/kg was injected into abdominal cavity. The cerebrum was sectioned coronally into 5 pieces of 2 mm thickness. The sections of the cerebrum were immediately stained with 2% TCC (Sigma-Aldrich., USA) in 6-well plates for 20 minutes. The sections were then transferred in paraformaldehyde. After 24 hours, each section was photographed and the infarction size was measured using the Image J (NIH, USA) software. After the fixation of the brain slices, the digital camera was taken out, and the area of infarct area was calculated by Image J image analysis software (dyeing weakened area of middle cerebral artery). In order to offset the error of the infarct volume caused by edema after cerebral infarction, using the indirect infarct volume method proposed by Lv et al. [15] measured the area of the ipsilateral noninfarct area and the contralateral area of the infarct in each slice of the brain, respectively. The infarct volume was expressed as the percentage of infarcted volume in the total volume of the contralateral hemisphere, which is calculated by the formula, (contralateral volume of infarct − ipsilateral noninfarct volume)/contralateral volume of infarct × 100%.

### 2.5. Neurological Deficit Scores

In accordance with Bederson's method [16], neurological deficits were scored and recorded 24 hours after model establishment. The scoring criteria are as follows: score 0: no symptoms of nerve injury; score 1: when lifting the tail, the contralateral forelimb of the lesion cannot be completely straightened; score 2: circling to the paralyzed side during walking; score 3: falling to the contralateral side of the lesion; score 4: cannot walk spontaneously. Neurological symptoms were scored by single blind method. That is, the observer was blinded to group assignment.

### 2.6. Histopathological Changes in the Brain Observed Using Hematoxylin-Eosin Staining

At 24 hours after MCAO, five rats were taken from each group, by intraperitoneal injection of 2% pentobarbital sodium anesthesia, eyeball bleeding put to death, and fixed brain tissue immersed into 10% formaldehyde. After 48 h, they were dehydrated in 70%, 80%, 90%, 95%, and 100% alcohol, respectively. Xylene transparent was impregnated, paraffin embedded, sliced, dewaxed, and hematoxylin stained for 3 minutes. The differentiation of hydrochloric acid and ethanol was for 3–5 seconds and eosin for 1 minute. The sections were rehydrated through a series of decreasing concentration of ethanol, dewaxed in xylene, and mounted with neutral gum. Each brain tissue observed 4 to 6 visual fields from cerebral cortex and hippocampus under microscope.

### 2.7. Neuronal Cells in the Brain as Determined Using Hematoxylin-Eosin Staining and Terminal Deoxynucleotidyl Transferase dUTP Nick End Labeling (TUNEL)

Neuronal apoptosis was detected with a TUNEL kit (No. 11684817910; Roche, USA) in strict accordance with the instruction. Five rat hippocampus were taken from each group, dehydrated, embedded, and sliced into sections. These sections were incubated with 0.9% NaCl for 5 minutes and washed twice with phosphate buffered saline (PBS). After removal of the solution, sections were incubated with biotinylated nucleotides and terminal deoxynucleotidyl transferase and covered with plastic cover at 37°C for 60 minutes. After washing with PBS, sections were blocked with 0.3% H_2_O_2_, washed three times with PBS, and incubated with streptavidin peroxidase labeled with horseradish peroxidase (HRP) for 30 minutes at room temperature. After washing with PBS, each brain tissue observed 4 to 6 visual fields from cerebral cortex and hippocampus under microscope.

### 2.8. NO, MDA, GSH-Px, and SOD in Brain Tissue and Brain Injury Markers S-100*β* and NSE Contents as Measured with Enzyme Linked Immunosorbent Assay (ELISA)

Five rat brain tissues were taken from each group, and each sample was measured 3 times for ELISA analysis. ELISA kit was used to examine NO (KGE001, R & D), MDA (CEA597Ge, USCN), GSH-Px (CEA294Ge, USCN) and SOD (SES134Ra, USCN), S-100*β* (SEA012Ra, USCN), and NSE (SEA537Ra, USCN) contents in accordance with the instruction. 100 *μ*ll standard and l00 *μ*l diluted specimens were added in corresponding reaction plate wells in order at 37°C for 30 minutes. After the plate was washed, 100 *μ*l checked samples were added in each well at 37°C for 2 hours. After washing the plate, l00 *μ*l HRP-labeled secondary antibody was added in each well at 37°C for 30 minutes. After washing the plate, the chromogenic solution A and the chromogenic solution B (each 50 *μ*l) were added in the dark for 15 minutes. The stop buffer 50 *μ*l was then added. Optical density (OD) values were measured at 450 nm using a microplate reader (EXL808, USA). Standard curves were drawn. The corresponding concentration of the sample was obtained according to the curve equation.

### 2.9. Western Blot Assay

Five rat brain tissues were taken from each group for western blot assay. Hippocampus tissue was treated with precooling tissue lysate and centrifuged at 12000 rpm for 30 minutes. Total protein was extracted from the supernatant and subjected to sodium dodecyl sulfate polyacrylamide gel electrophoresis and then transferred to the membrane using semidry method. The membrane was blocked with confining liquid for 2 hours, incubated with BCl_2_ (ab32124, Abcam, USA), Bax (ab32503, Abcam, USA), Caspase-3 (ab13847, Abcam, USA), PI3K (ab86714, Abcam, USA), p-AKT (ab38449, Abcam, USA), and eNOS (ab5589, Abcam, USA) primary antibody at 4°C overnight, and washed three times with TBST. Afterwards, the membrane was incubated with secondary antibody for 1 hour, washed four times with TBST, and visualized with enhanced chemiluminescence kit. Images were obtained with a gel imaging system. Gray values were measured with Quantity One software.

### 2.10. Expressions of PI3K, p-AKT, and eNOS Were Detected by Immunohistochemistry

Five rat brain tissues were taken from each group for* immunohistochemistry*. The brain tissue was fixed in 4% paraformaldehyde, embedded in paraffin, sliced at the thickness of about 4 *μ*m, and then placed in 58–60°C oven for 30–60 minutes. Slices were dewaxed for 15 minutes, rinsed with distilled water three times, and immersed in 0.01 M citrate buffer (pH 6.0). Then slices were heated to boiling, cutting off the power, every 5–10 minutes, and rinsed with 0.02 M citrate buffer once or twice. Tissue was incubated with normal rabbit blocking solution for 20 minutes at room temperature and with PI3K, p-AKT, and eNOS antibody at 37°C for 1 hour, followed by another 0.02 M PBS washing for 2 minutes, a total of three times; then tissue was added with biotinylated goat anti-rabbit IgG at 37°C for 20 minutes; rinsed with 0.02 M PBS for 2 minutes, totally three times; incubated with reagent SABC at 37°C for 20 minutes and rinsed with 0.02 M PBS for 5 minutes, totally four times; and then stained with DAB, counterstained with hematoxylin, dehydrated, permeabilized, mounted, and observed under the microscope (*n* = 3 rats per group and 5 slides per rat).

### 2.11. Statistical Analysis

The data were analyzed using SPSS 19.0 software and expressed as mean ± standard deviation. Intergroup difference was compared using one-way analysis of variance. Intragroup difference was compared using repeated measures analysis of variance. A value of *P* < 0.05 was considered statistically significant.

## 3. Results

### 3.1. EGCG Reduces Neurological Impairment in Rats after MCAO

**Figure 1:**
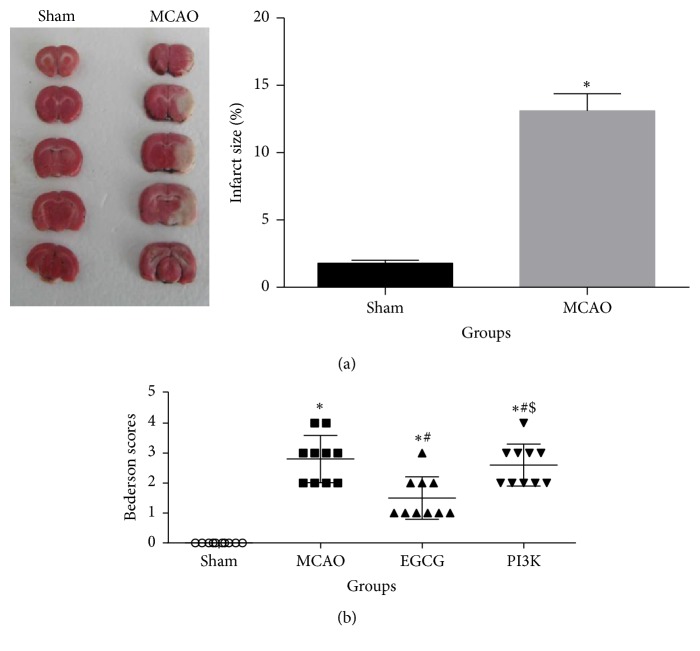
*TCC staining and neurological deficit scores in rats*. (a) TCC staining. (b) Neurological deficit scores. Compared with the sham group, ^*∗*^*P* < 0.05. Compared with the MCAO group, ^#^*P* < 0.05; compared with the EGCG group, ^$^*P* < 0.05.

**Figure 2:**
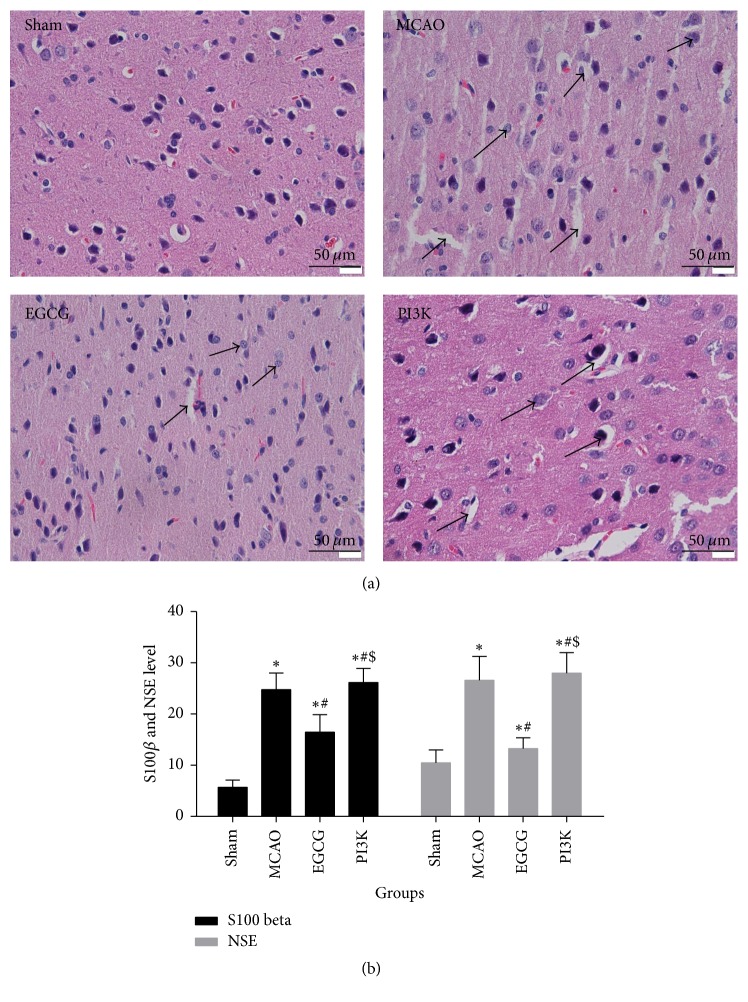
*Brain injury in rats after MCAO*. EGCG can reduce brain tissue injury and inhibit the expression of S100*β* and NSE. The protective effect of EGCG decreases after adding PI3K inhibitor. (a) HE staining of brain (×200) from rats in each group. Bar, 50 *μ*m. (b) S100*β* and NES levels measured by ELISA. Compared with the sham group, ^*∗*^*P* < 0.05. Compared with the MCAO group, ^#^*P* < 0.05. Compared with the EGCG group, ^$^*P* < 0.05.

TTC staining of the individual brains demonstrated that the MCAO group had significantly larger infarct size of the cortex than the sham group (Figure 1). Our MCAO model was successful. Neurological deficits were assessed with Bederson scores. Results are shown in Figure 2. Neurological deficit scores were significantly higher in the MCAO group than in the sham group, indicating that MCAO caused obvious neurological deficits. Compared with the MCAO group, neurological deficit scores were significantly lower in the EGCG group (*P* < 0.05). After intervention with PI3K inhibitor, neurological deficit scores were not significantly different as compared with the MCAO group (*P* > 0.05). These findings suggest that EGCG has a protective effect on neurological impairment in rats, and its mechanism is related to PI3K.

### 3.2. EGCG Mitigates Brain Injury in Rats after MCAO

In the sham group, brain tissue was normal and cell boundary was distinct in the cortex. In the MCAO group, neuronal injury was visible in the cortex; pyramidal cells were not regularly arranged and swollen; cell membrane structure was not distinct; neuronal degeneration, pyknosis in some neurons, and unclear structure were observed (Figure 2(a)). ELISA results demonstrated S-100*β* and NSE contents (Figure 2(b)). Results were significantly increased (versus sham group; *P* < 0.05). In the EGCG group, nerve cells were regularly distributed. Neuronal degeneration and necrosis lessened. Pyknosis was seen occasionally. S-100*β* and NSE contents decreased (versus MCAO group; *P* < 0.05). In the PI3K group, the protective effect of EGCG on MCAO model could be reversed. Neuronal injury was visible. The infarct area increased. S-100*β* and NSE contents increased.

### 3.3. EGCG Inhibits Oxidative Stress after MCAO

**Figure 3:**
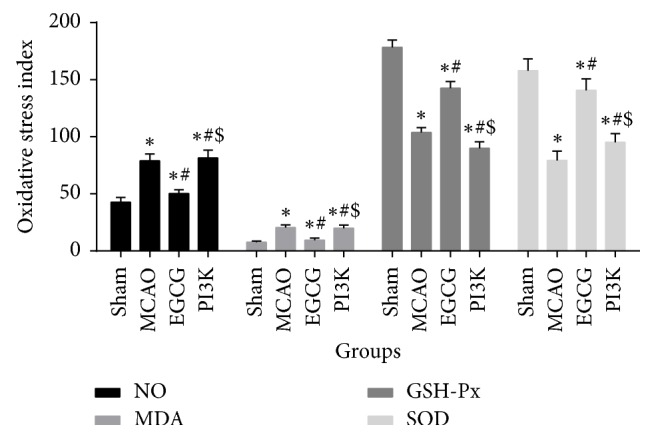
*EGCG inhibits oxidative stress*. EGCG can increase GSH-Px and SOD expression and reduce NO and MDA levels. The levels of GSH-Px and SOD decrease and NO and MDA increase after adding PI3K inhibitor. Compared with the sham group, ^*∗*^*P* < 0.05. Compared with the MCAO group, ^#^*P* < 0.05. Compared with the EGCG group, ^$^*P* < 0.05.

Compared with the sham group, NO and MDA significantly increased, but GSH-Px and SOD contents diminished after model establishment; significant differences were found between the two groups (*P* < 0.05). After intraperitoneal injection with EGCG, oxidative stress factor contents reduced in the brain tissue, but PI3K could effectively suppress the intervention of EGCG on oxidative stress (Figure 3). It is thus clear that EGCG plays an antioxidant role in MCAO model.

### 3.4. EGCG Mitigates Neuronal Apoptosis Induced by MCAO

**Figure 4:**
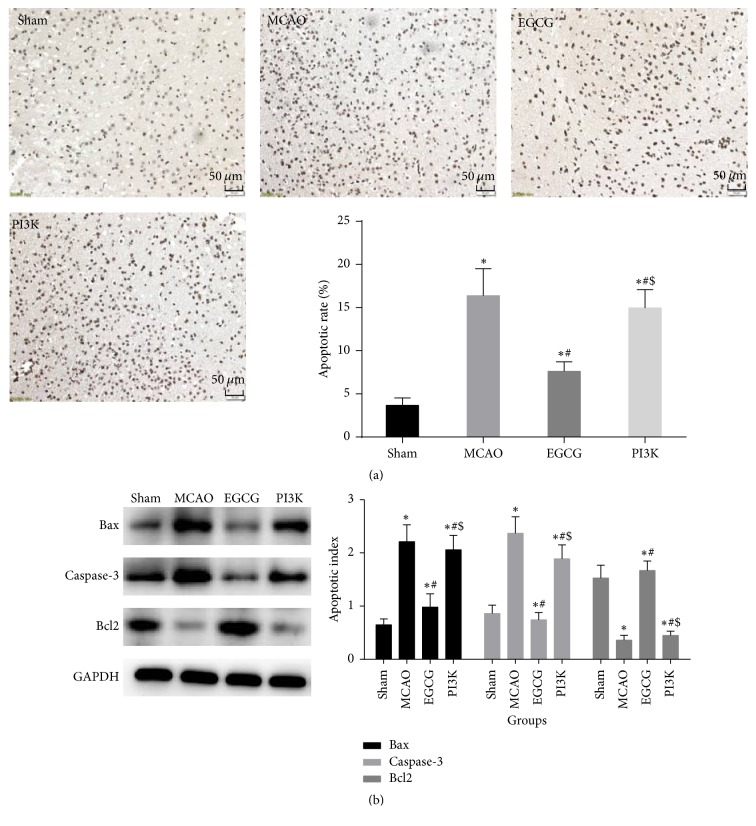
*EGCG attenuates neuronal apoptosis*. EGCG attenuates neuronal apoptosis, inhibits the expression of Bax and Caspase-3, and increases the expression of Bcl2, after adding PI3K inhibitor the protective effect of EGCG decreases. (a) Apoptosis positive cells by TUNEL in brain (×200) from rats in each group by TUNEL. Bar, 50 *μ*m. (b) Apoptosis-relative protein by western blot assay. Compared with the sham group, ^*∗*^*P* < 0.05. Compared with the MCAO group, ^#^*P* < 0.05. Compared with the EGCG group, ^$^*P* < 0.05.

Compared with the sham group, neuronal apoptosis was noticeable and apoptotic rate increased in the MCAO group (Figure 4(a)). Bcl-2 expression significantly reduced, but Caspase-3 and Bax expression significantly increased (Figure 4(b); *P* < 0.05). Compared with the MCAO group, apoptotic rate of neurons significantly diminished; Bcl-2 expression significantly increased; Caspase-3 and Bax expression significantly decreased in the EGCG group (*P* < 0.05). Compared with the EGCG group, the apoptotic rate of neurons significantly increased; Bcl-2 expression significantly reduced and Caspase-3 and Bax expression significantly increased in PI3K group (*P* < 0.05).

### 3.5. EGCG Lessens Brain Injury in Rats after MCAO through PI3K/AKT/eNOS Signaling Pathway

**Figure 5:**
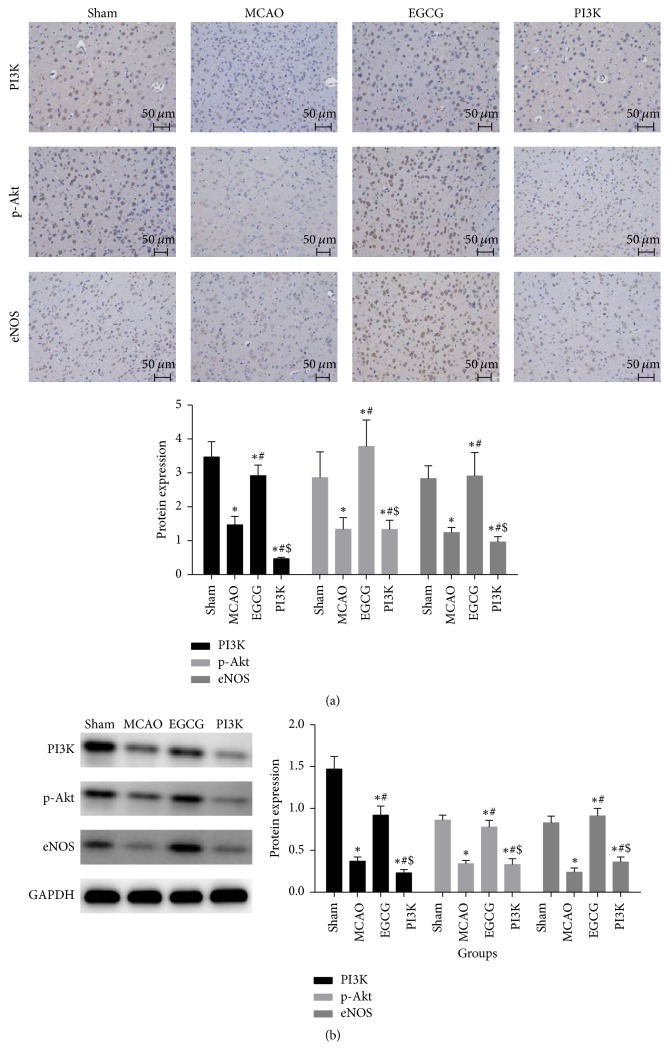
*EGCG attenuates brain injury after MCAO through PI3K/AKT/eNOS signaling pathway*. After adding PI3K inhibitor, the expression of PI3K, p-Akt, and eNOS significantly decreases. (a) The expression of PI3K, p-Akt, and eNOS detected by immunohistochemistry of brain (×200) from rats in each group. Bar, 50 *μ*m. (b) The expression of PI3K, p-Akt, and eNOS detected by western blot assay. Compared with the sham group, ^*∗*^*P* < 0.05. Compared with the MCAO group, ^#^*P* < 0.05. Compared with the EGCG group, ^$^*P* < 0.05.

PI3K, p-AKT, and eNOS protein expressions were measured using immunohistochemistry (Figure 5(a)) and western blot assay (Figure 5(b)). Compared with the sham group, eNOS, PI3K, and p-AKT expressions significantly decreased in the MCAO group after model establishment (*P* < 0.05). Compared with the MCAO group, eNOS, PI3K, and p-AKT expression significantly increased in the EGCG group (*P* < 0.05). Compared with the EGCG group, eNOS, PI3K, and p-AKT expressions significantly decreased in the PI3K group (*P* < 0.05).

## 4. Discussion

The lesion center of cerebral infarction presents ischemic necrosis, surrounded by ischemic penumbra. It is a hot issue in the treatment of cerebral infarction to increase the collateral circulation, save the dying brain cells in the penumbra, strengthen neuroprotection, and reduce the apoptosis of brain cells. Results from this study confirmed that EGCG could lessen neurological impairment in rats after MCAO, mitigate MCAO-induced brain injury, suppress inflammatory factor expression induced by MCAO, reduce proapoptotic protein expression, and alter protein expression in PI3K, p-AKT, and eNOS signaling pathway. Results indicated that EGCG had protective effect on brain injury induced by MCAO in rats, possiblythrough regulating PI3K/AKT/eNOS signaling pathway.

EGCG has obvious effect on the prevention and treatment of antioxidant, anti-inflammation, antivirus, cardiovascular disease, cancer, obesity, diabetes, endometritis, ulcerative colitis and autoimmune disease, and the regulation of lipid metabolism [17–19]. At 3 hours after ischemia, intraperitoneal injection of EGCG could mitigate neuronal injury and played a neuroprotective role in gerbil models[20]. After intraperitoneal injection of different concentrations of EGCG, Lee et al. [21] found that 20 mg/kg can effectively reduce MDA level and decrease brain edema and cerebral infarction area in models of focal cerebral ischemia reperfusion. Rahman et al. [22] established SDrat models of transient regional cerebral ischemia by intraperitoneal injection with EGCG20 mg/kg; 72 hours after surgery; they found that EGCG could reduce cerebral infarction area and was not toxic to liver, kidney, and spleen. In this study, rat models of MCAO were intervened with EGCG; results showed that EGCG obviously diminished neurological deficit score, improved histopathological damage, and inhibited oxidative stress and apoptosis. EGCG could reduce neurological impairment and relieve brain injury induced by MCAO in rats.

Lipid peroxidation induced by oxygen free radicals is one of the important mechanisms of cerebral ischemia/reperfusion injury after cerebral ischemia. A large number of oxygen free radicals are produced during ischemia/reperfusion, resulting in protein and lipid peroxidation, especially polyunsaturated fatty acid peroxidation on cellular lipids; these injure cell membranes, organelles, tissue structure, and function and lead to endothelial cell dysfunction [23]. EGCG has high antioxidant activity, which is 25 times that of vitamin E and more than 100 times that of vitamin C. EGCG can protect cells and DNA against injury [24]. Wang and Tian [25] used EGCG for neurodegenerative diseases induced by homocysteine. Their results confirmed that EGCG could improve oxidative stress, nerve inflammation, and nerve cell apoptosis and improve brain injury induced by homocysteine. Our results demonstrated that EGCG could reduce MDA and NO and upregulate GSH-Px and SOD contents, suggesting that protective effect of EGCG may be associated with the inhibition of lipid peroxidation and the improvement of antioxidant enzyme activity.

PI3K/Akt signaling pathway is an important pathway to promote cell survival. Activated PI3K could further activate downstream protein kinase Akt. Activated Akt can phosphorylate Bax, inactivate Bax, and block Bax and Bcl-2 to form dimers, thereby resulting in Bcl-2 dissociation to play antiapoptotic effect [26]. Chen et al. [27] investigated the ischemia/reperfusion mechanism in MCAO rat models and verified that Bcl-2 expression reduced, but Caspase-3 and Bax expression increased. Lv et al. [15] confirmed that *δ*-opioid receptor agonist improved neuronal apoptosis after cerebral ischemia/reperfusion through PI3K-Akt activation pathway. In the present study, MCAO models were treated with EGCG, and PI3K antagonist LY294002 was injected in the brain. Our results demonstrated that EGCG suppressed neuronal apoptosis, increased Bcl-2 expression, decreased Caspase-3 and Bax expression, and upregulated eNOS, PI3K, and p-AKT expression. These changes were eliminated by LY294002. These findings suggested that EGCG improved neuronal apoptosis in MCAO rats probably through PI3K/Akt/eNOS signaling pathway. Activation of the PI3K/Akt signaling pathway may be an important method of treating neurodegenerative diseases.

In summary, EGCG could mitigate neurological impairment and brain injury induced by MCAO, suppress the expression of oxidative stress factors, and reduce neuronal apoptosis and later protein expression in PI3K, p-AKT and eNOS signaling pathway. Our results indicated that EGCG had protective effects on MCAO-induced brain injury in rats, possibly via regulating the PI3K/AKT/eNOS signaling pathway.
